# Occlusion Robust Cognitive Engagement Detection in Real-World Classroom

**DOI:** 10.3390/s24113609

**Published:** 2024-06-03

**Authors:** Guangrun Xiao, Qi Xu, Yantao Wei, Huang Yao, Qingtang Liu

**Affiliations:** 1 School of Mechanical Engineering, Hubei University of Arts and Science, Xiangyang 441053, China; xiaoguangrun@hbuas.edu.cn; 2 Hubei Key Laboratory of Digital Education, Central China Normal University, Wuhan 430079, China; xuqi@mails.ccnu.edu.cn (Q.X.); liuqtang@ccnu.edu.cn (Q.L.); 3 Faculty of Artificial Intelligence in Education, Central China Normal University, Wuhan 430079, China; yaohuang@ccnu.edu.cn

**Keywords:** cognitive engagement, automatic detection, real-world classroom, YOLO

## Abstract

Cognitive engagement involves mental and physical involvement, with observable behaviors as indicators. Automatically measuring cognitive engagement can offer valuable insights for instructors. However, object occlusion, inter-class similarity, and intra-class variance make designing an effective detection method challenging. To deal with these problems, we propose the Object-Enhanced–You Only Look Once version 8 nano (OE-YOLOv8n) model. This model employs the YOLOv8n framework with an improved Inner Minimum Point Distance Intersection over Union (IMPDIoU) Loss to detect cognitive engagement. To evaluate the proposed methodology, we construct a real-world Students’ Cognitive Engagement (SCE) dataset. Extensive experiments on the self-built dataset show the superior performance of the proposed model, which improves the detection performance of the five distinct classes with a precision of 92.5%.

## 1. Introduction

In order for students to successfully learn during classroom instruction, it is essential that they pay attention and actively engage with the learning content. Research has shown that students demonstrate varying levels of cognitive engagement even within the same instructional settings (e.g., [1]). Therefore, it is crucial for teachers to ascertain whether and to what extent students are focusing on the learning materials [2]. According to the Interactive, Constructive, Active, and Passive (ICAP) framework [3], students exhibit enhanced learning outcomes when participating in active or interactive learning activities as opposed to passively receiving information. Prior studies have indicated that students display attention-related behaviors that are indicative of their underlying cognitive processes [3,4,5]. Engagement with instructional content is considered active when it involves overt motoric actions or physical manipulation. However, measuring cognitive engagement through observing changes in students’ behavior can be time-consuming and labor-intensive (e.g., [6,7]), especially in long-duration courses and large face-to-face classes. Thus, there is a need for additional measures that could provide high-efficiency and low-cost information on cognitive engagement during real-world classroom learning.

The You Only Look Once (YOLO) series is a useful method for object detection tasks. These methods have been demonstrated to be applicable for detecting students’ in-class behaviors (e.g., sleeping, drinking, yawning, etc. [8]). When considering the ICAP framework, YOLO applied to cognitive engagement detection offers three advantages [2,9]: (1) using low-cost visual data, (2) tracking the location of students’ behaviors, (3) efficiently establishing the relationship between behaviors and cognitive engagement, and (4) measuring students’ cognitive levels.

However, the behaviors’ occlusion problem in the real-world classroom makes cognitive engagement detection extremely challenging. Furthermore, it is a big challenge for students to maintain a state of constructive or interactive engagement, resulting in a scarcity of labeled samples. Therefore, this study aims to develop an improved object-enhanced method utilizing YOLO version 8 nano (YOLOv8n) models to monitor students’ cognitive engagement. This method aims to alleviate small-scale dataset issues through data augmentation and solve occluded behavior problems through IoU loss enhancement. The primary contributions of this research are as follows:

- The Students’ Cognitive Engagement (SCE) dataset in a real-world classroom is built. In contrast to experiment-induced behaviors, this method non-invasively collects visual data of students, which serve as valuable input for training automatic detection models in authentic classroom settings.
- The Object-Enhanced–YOLOv8n (OE-YOLOv8n) method is proposed to detect students’ cognitive engagement in real-world scenes. First, it enhances the computation of easily occluded behaviors. Second, it enhanced the small-scale cognitive engagement data.
- The OE-YOLOv8n method uses Mosaic and Cutout methods to enhance the real-world cognitive engagement data. Subsequently, it leverages the Inner Minimum Point Distance Intersection over Union (IMPDIoU) loss function, refining the key point distance between the potentially occluded predicted box and its corresponding ground truth box.

## 2. Related Works

### 2.1. Cognitive Engagement Detection

Cognitive engagement involves both mental and physical participation. Previous studies indicate that self-reports can capture psychological information on cognitive engagement (e.g., [6,7]). However, there are still concerns about the reliability and validity of self-reporting [10,11]. Another widely used method in the educational community is classroom observation. This approach accounts for the explicit information of cognitive engagement, but it is time-consuming and challenging to apply to long and large classes. Goldberg et al. [2] suggested that automated detected approaches are more efficient, accurate, and time-saving. Indeed, recent studies have characterized cognitive engagement as detecting students’ voices [12], texts [13], behaviors [14], etc. Such a perspective might provide a more precise and traceable insight into the visual components of cognitive engagement [15]. Currently, there are many databases on cognitive engagement. However, existing datasets like MOOC learners’ discussion (text data, [16]) and the RECOLA Dataset (speech/multimodal data, [12,17]) are not well suited for real-world learning. In classrooms, teachers are usually the initiators of instruction, and students’ speech data are limited and sparse. Visual data, on the other hand, contain explicit information about students’ classroom expressions and feedback. Therefore, visual data are suitable for detecting cognitive engagement. The ICAPD dataset [9] is an example of an image-based cognitive engagement dataset, but its small scale makes it challenging to ensure the robustness of models. Target detection technology (e.g., YOLO [18]) is a popular automatic assessment method for students and their behaviors. Utilizing YOLO with visual data enhances the efficiency and accuracy of cognitive engagement detection.

The YOLO network concerns the statistical probability distribution of the target area. When different categories of target regions exhibit sufficient discriminability, they can be used for detecting various behaviors. For example, Chen and Guan [19] used related YOLO networks to detect the teacher’s behaviors (i.e., explaining questions, pointing to the projection, no hand gestures, gesturing with both hands, head down and operating, walking around, writing on the blackboard, and guiding students to raise their hand) and the students’ behaviors (i.e., looking up, head dropping, hand raising, standing up, lying on the desk). However, the probability distribution of cognitive engagement regions in images poses a challenging task when using YOLO, as the explicit behavioral states of cognitive engagement lack a clear definition. To achieve this, we developed the ICAPD framework to simulate the distribution of behaviors as targets. This is because different behaviors have distinct distributions, and by treating them as targets, we can leverage the capabilities of the YOLO network to detect and localize these behaviors accurately. By considering behaviors as targets, we can define specific regions of interest within an image where these behaviors are likely to occur. The YOLO network then learns to predict bounding boxes and associated probabilities for these behavior targets. This approach allows us to effectively model and detect complex cognitive engagement, even in scenarios where occlusion or limited pixel presence may be present. YOLOv8 is a recent version of the YOLO series. This version incorporates an anchor-free mechanism within its head module. So, it can directly predict the behavior’s center point and width-to-height ratio. Additionally, YOLOv8’s loss function incorporates both classification and regression branches. The classification branch uses the standard Binary Cross-Entropy (BCE) loss function, while the regression branch utilizes Distribution Focal Loss combined with Complete Intersection over Union (DFL-CIoU) Loss. These approaches enable effective measurement of both the location of behaviors and the classes of cognitive engagement. Therefore, applying YOLO technology to cognitive engagement detection tasks is feasible. Because cognitive engagement can be characterized as low-cost visual data, automatic cognitive engagement detection will make learning analysis more efficient.

### 2.2. Small-Scale Object Detection with YOLO

**Figure 1:**
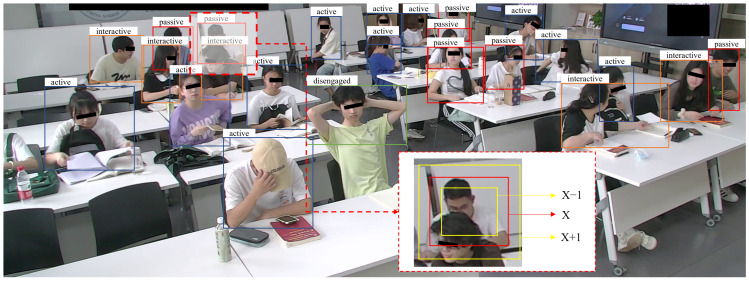
Student learning scene in a real-world classroom. The red boxes indicate the ground truth bounding boxes corresponding to cognitive engagement, whereas the yellow boxes represent the predicted bounding boxes for cognitive engagement.

In a real-world classroom, a small-scale sample problem constitutes the students’ cognitive engagement level. In comparison to laboratory settings, real-world scenarios exhibit greater complexity. While lecture courses can provide many samples of passive engagement, high-level engagement samples are often limited. Although collaborative or discussion-based classes can elicit high-level engagement data, they are still limited for model training purposes. The small-sample issue in cognitive engagement detection is attributed to data availability and annotation complexity [3]. Firstly, acquiring a large dataset for cognitive engagement necessitates prolonged classroom sessions and the participation of multiple students. However, extended student engagement can lead to fatigue. Secondly, annotating cognitive engagement based on behavior requires specialized training and expertise. It is time-consuming and resource-intensive. Furthermore, not all behaviors in an image are usable (e.g., as shown in Figure 1). Addressing how to train a highly robust model on a small-scale dataset is a critical issue that needs to be tackled.

Previous studies have shown that data augmentation is an effective method to alleviate small-scale sample problems, as it allows for more complex representations of data in classes with all samples. This facilitates the network to learn the data distribution of the dataset better. Image processing techniques are commonly used to augment datasets and optimize image quality, and they can be categorized into three main types: geometric transformations, color transformations, and pixel transformations [20].

Geometric transformation techniques, such as image cropping and scaling, image shifting and padding, and image flipping and rotation, are not meaningful for text recognition tasks. However, they can effectively address datasets with positional biases. Color transformation, such as color space conversion, is a highly effective way of extracting color features [21]. However, selecting the appropriate color space transformation to enhance model performance remains challenging. Pixel transformation techniques (e.g., noise, blur, pixel fusion, and information deletion) provide a novel solution for image generation tasks, especially the Cutout technique [22,23]. This technique randomly applies square paths of a certain size at random positions on the image to create a 0-mask crop. The benefit is that it avoids unnatural artifacts caused by image blending and can enhance the model’s classification performance.

Owing to the challenge posed by small-scale sampling in real-world classrooms, geometric transformation techniques can enable the detecting models to pay more attention to complex behavioral features. In contrast, pixel transformation techniques can effectively mitigate overfitting problems. However, almost all of these transformations involve a distortion magnitude parameter. The combination of augmentations, encompassing flipping, cropping, color shifts, and random erasing, can lead to significantly increased dataset sizes. The efficacy of these transformations still requires experimental validation in real classroom environments.

### 2.3. Occluded Object Detection with YOLO

The YOLO method in cognitive engagement detection tasks faces some insurmountable problems, such as Occluded Objects (e.g., as shown in Figure 1, individuals in the front row occlude the behaviors exhibited by those in the back row, or the background impedes the visibility of the behavior). The density of students in the image causes the occlusion phenomenon. In an image, a target behavior may be obscured by a non-target background or another target behavior. In these scenarios, the effective behavioral features in the bounding box are reduced, leading to a decrease in YOLO performance. Previous studies have demonstrated that the design of the IoU loss function can impact the accuracy of object detection [24]. The IoU loss for bounding box regression exhibits significant sensitivity differences across objects of different scales. Therefore, based on the information of the bounding box (e.g., shape, aspect ratio, etc.), the design of an appropriate IoU loss function for detection has widely been of concern.

Currently, the design ideas for improving IoU loss functions include GIoU [25], DIoU [26], CIoU, EIoU [27] etc. However, in Figure 1, marked with dashed lines and magnified, the occlusion behavior labeled with red lines is classified as a passive category. Interestingly, under different IoU loss functions (i.e., GIoU, DIoU, CIoU, and EIoU), both larger and smaller red bounding box (i.e., labeled with yellow lines in Figure 1) predictions yield the same results [28]. However, the larger yellow bounding box contains more noise information (i.e., the head of the front-row student). Assigning it a higher loss weight may help the model learn better. The IoU loss function, which considers the overlapping area, the distance between center points, and deviations in width and height, would be more advantageous for the learning of models. This is particularly important when distinguishing between bounding boxes with the same aspect ratio but different sizes or positions.

Previous research has suggested that incorporating smaller auxiliary bounding box calculation losses during model training can have a positive impact on the regression of high-IoU samples. Conversely, low-IoU samples exhibit the opposite effect [27]. However, ensuring that the aspect ratios of the behaviors’ bounding boxes are the same is challenging. Zhang et al. [29] proposed addressing this issue by employing auxiliary bounding boxes of varying scales tailored to different datasets. Based on the above, designing improved IoU loss functions is a good solution for cognitive engagement tasks. It requires simultaneous consideration of enhancing limited features within occluded target bounding boxes and adaptively adjusting their aspect ratios.

## 3. The Proposed Method

### 3.1. Overall Architecture

**Figure 2:**
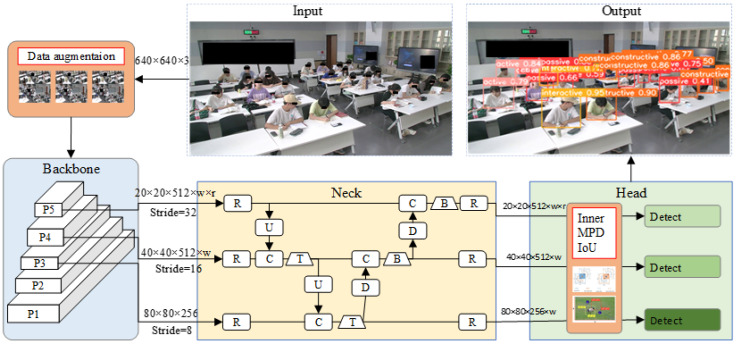
The structure of the improved OE-YOLOv8n model for cognitive engagement detection.

Here, an improved OE-YOLOv8n model is proposed for the detection of students’ cognitive engagement in real-world classrooms. The improved components of our improved OE-YOLOv8 model are the IMPDIoU loss function and cognitive engagement data processing. The comprehensive block diagram delineating the entire methodology is depicted in Figure 2. The improved OE-YOLOv8n model can detect complex cognitive engagement by target enhancement components, which may involve occlusion behaviors or occur in students with limited pixels. The components consist of an occlusion behavior enhancement loss component and an effective data augmentation component. This loss component uses the IMPDIoU loss function. This method minimizes the distance between the top-left and bottom-right points of the predicted bounding box and the ground truth bounding box by considering the relevant loss factors (i.e., overlapping or non-overlapping area, central point distance, and deviation in width and height). The data augmentation component employs the Mosaic and Cutout techniques. The Mosaic technique enhances the learning scene information to focus the model more on the targeted cognitive engagement. The Cutout technique enhances the model’s localization ability by adding information from other samples in the cut region.

Firstly, the proposed method performs data enhancement on the training samples. Next, the backbone layer is utilized to extract key features of student behavior. These key features, obtained at different scales, are fused in the neck layer. Finally, under the improved IoU loss function, the head layer outputs five categories of the students’ cognitive engagement during learning. Detailed methodologies are expounded upon in Section 3.2 and Section 3.3, where we introduce a data-augmented method and an enhanced IoU loss function, respectively.

### 3.2. OE-YOLOv8n with Mosaic and Cutout Data Augmentation

**Figure 3:**
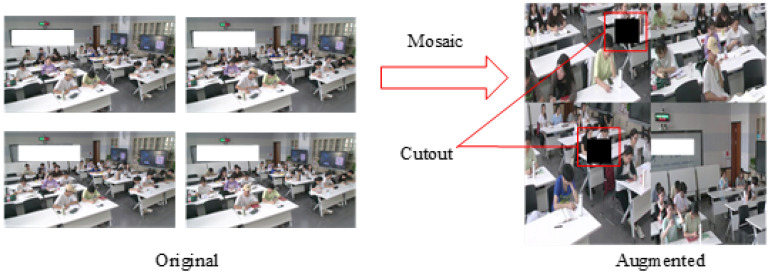
Mosaic and Cutout data augmentation.

We employed combined augmentations, including geometric transformation techniques and pixel transformation techniques, as shown in Figure 3. (1) We utilized the Mosaic method for geometric transformation techniques. We combined four images, each with its corresponding bounding boxes, into a single new image. This new image and its corresponding bounding boxes were then fed into the neural network for learning. Essentially, we simultaneously input four images for learning, which yielded excellent results for detecting small objects. (2) We drew inspiration from the Cutout technique for pixel transformation techniques. We randomly covered a portion of the image, equivalent to 10% of the original image size, with two black boxes. This means that we cut out certain regions of the samples and filled them with zero pixel values. The purpose was to enhance the model’s robustness to occluded situations.

### 3.3. OE-YOLOv8n with an IMPDIoU Loss Function

Then, the predicted bounding box and its respective categories were emitted, and the targets were annotated within the original image to facilitate the detection of student objects present. The loss associated with the target score was calculated by the BCE Logits loss function, while the class probability score was assessed through the cross-entropy loss function (BCE cls loss). Inspired by the geometric properties of bounding boxes, we used IMPDIoU to replace IoU [28,29] as the model’s loss function. It minimizes the distance between the predicted and ground truth bounding boxes.

In order to further address the limited generalization capability and slow convergence speed exhibited by the existing MPDIoU loss function in different detection tasks, a scale factor ratio was incorporated to modulate the scale size of auxiliary bounding boxes, thereby accelerating the bounding box regression process.

Let $B^{gt}$ denote the ground truth box and $B^{pd}$ denote the predicted bounding box. The coordinates in the top-left corner, bottom-right corner, and center point of the ground truth box are denoted as $\left(x_{1}^{gt},y_{1}^{gt}\right)$, $\left(x_{2}^{gt},y_{2}^{gt}\right)$, and $\left(x_{c}^{gt},y_{c}^{gt}\right)$, respectively. Similarly, the coordinates of the corresponding points in the predicted bounding box are represented by $\left(x_{1}^{pd},y_{1}^{pd}\right)$, $\left(x_{2}^{pd},y_{2}^{pd}\right)$, and $\left(x_{c}^{pd},y_{c}^{pd}\right)$. The dimensions of the ground truth box, namely its width and height, are indicated by $w^{gt}$ and $h^{gt}$, respectively. In a analogous manner, the width and height of the predicted bounding box, as well as those of the input image, are denoted by $w^{pd}$, $h^{pd}$, *w*, and *h*. The variable “ratio” represents the scale factor, ranging from 0.5 to 1.5. The IMPDIoU is defined in Equations (1)–(9):

(1) $$\begin{array}{c} b_{l}^{gt}=x_{c}^{gt}-\frac{w^{gt}\times ratio}{2},b_{r}^{gt}=x_{c}^{gt}+\frac{w^{gt}\times ratio}{2} \end{array}$$

(2) $$\begin{array}{c} b_{t}^{gt}=y_{c}^{gt}-\frac{h^{gt}\times ratio}{2},b_{b}^{gt}=y_{c}^{gt}+\frac{h^{gt}\times ratio}{2} \end{array}$$

(3) $$\begin{array}{c} b_{l}^{pd}=x_{c}^{pd}-\frac{w^{pd}\times ratio}{2},b_{r}^{pd}=x_{c}^{pd}+\frac{w^{pd}\times ratio}{2} \end{array}$$

(4) $$\begin{array}{c} b_{t}^{pd}=y_{c}^{pd}-\frac{h^{pd}\times ratio}{2},b_{b}^{pd}=y_{c}^{pd}+\frac{h^{pd}\times ratio}{2} \end{array}$$

(5) $$\begin{array}{cc} B^{gt}\cap B^{pd} & =(min\left(b_{r}^{gt},b_{r}^{pd}\right)-max\left(b_{l}^{gt},b_{l}^{pd}\right))\\ \; & \times \;(min\left(b_{b}^{gt},b_{b}^{pd}\right)-max\left(b_{t}^{gt},b_{t}^{pd}\right)) \end{array}$$

(6) $$\begin{array}{cc} B^{gt}\cup B^{pd} & =\left(w^{gt}\times h^{gt}\right)\times {\left(ratio\right)}^{2}\\ \; & +\left(w^{pd}\times h^{pd}\right)\times {\left(ratio\right)}^{2}-B^{gt}\cap B^{pd} \end{array}$$

(7) $$\begin{array}{c} d_{1}^{2}={\left(x_{1}^{bg}-x_{1}^{pd}\right)}^{2}+{\left(y_{1}^{bg}-y_{1}^{pd}\right)}^{2} \end{array}$$

(8) $$\begin{array}{c} d_{2}^{2}={\left(x_{2}^{bg}-x_{2}^{pd}\right)}^{2}+{\left(y_{2}^{bg}-y_{2}^{pd}\right)}^{2} \end{array}$$

(9) $$\begin{array}{c} IMPDIoU=\frac{B^{gt}\cap B^{pd}}{B^{gt}\cup B^{pd}}-\frac{d_{1}^{2}}{w^{2}+h^{2}}-\frac{d_{2}^{2}}{w^{2}+h^{2}} \end{array}$$

Thus, the loss function derived from IMPDIoU can be defined as follows:

(10) $$\begin{array}{c} L_{IMPDIoU}=1-IMPDIoU \end{array}$$

In comparison to the standard MPDIoU loss, when the ratio is below 1 and the auxiliary bounding boxes are smaller than the actual ones, the effective range of regression is reduced. Nonetheless, the gradient’s absolute value exceeds that of the MPDIoU loss, facilitating accelerated convergence for high-IoU samples. Conversely, when the ratio is above 1, the expanded scale of the auxiliary bounding boxes enhances the regression’s effective range, thereby improving the regression effect for low-IoU samples. The value of the ratio was established through the verification method employed in the subsequent experiment.

The outcomes of the comparative experiment involving various ratios within the IMPDIoU loss, as opposed to the standard MPDIoU loss, are detailed in Table 1. It is apparent that the loss function employed in this paper demonstrates a substantial enhancement across all detection metrics when contrasted with the standard MPDIoU loss. Furthermore, an observation can be made that with the increment of the ratio value for the dataset utilized in this study, there is a corresponding improvement in the detection outcomes. Notably, the two primary indicators, F1 and Map, both achieve their peak performance at a ratio of 1.4. Hence, a ratio of 1.4 was ultimately determined as the optimal choice for this paper.

## 4. Experimental Results and Discussion

### 4.1. SCE Dataset

**Figure 4:**
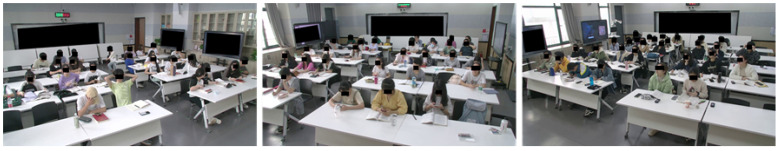
Three examples from the dataset.

Our SCE dataset not only considers the validity and discriminability of cognitive engagement categories but also takes into account the diversity and complexity of student populations, enabling a more effective explanation of the occurrence, changes, and maintenance of students’ cognitive engagement. The SCE dataset (Approval CNU-IRB-202305004a) is a real-world Students’ Cognitive Engagement dataset. SCE has several main features: a real-world classroom, cognitive engagement detection, an ICAPD annotation framework, 6566 images (6566 annotated), 86 students, 5 categories for students’ cognitive engagement, and 25–30 students per image. We used non-invasive cameras above the college classroom’s blackboard to collect data. The collected data are all spontaneous behaviors of students, thereby preserving the authenticity of classroom learning as much as possible. Figure 4 shows the examples from the dataset. A total of eight videos were collected from the different classes, each with a resolution of 1920×1080. Six videos last 45 min and two videos last 90 min. Each video contains 25–30 students. Then, automatic frame sampling was performed to obtain frames that are conducive to automated detection. And the images were extracted from video stream frames every three seconds. The extracted images were stored in the folder as samples to be labeled.

**Figure 5:**
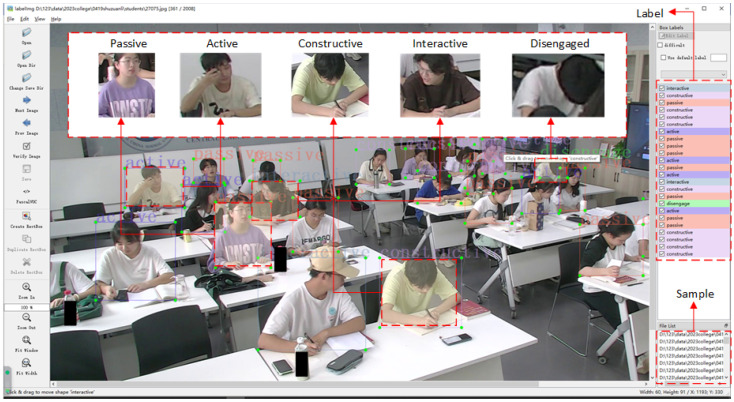
An example during labeling with the Labelimg tool.

**Figure 6:**
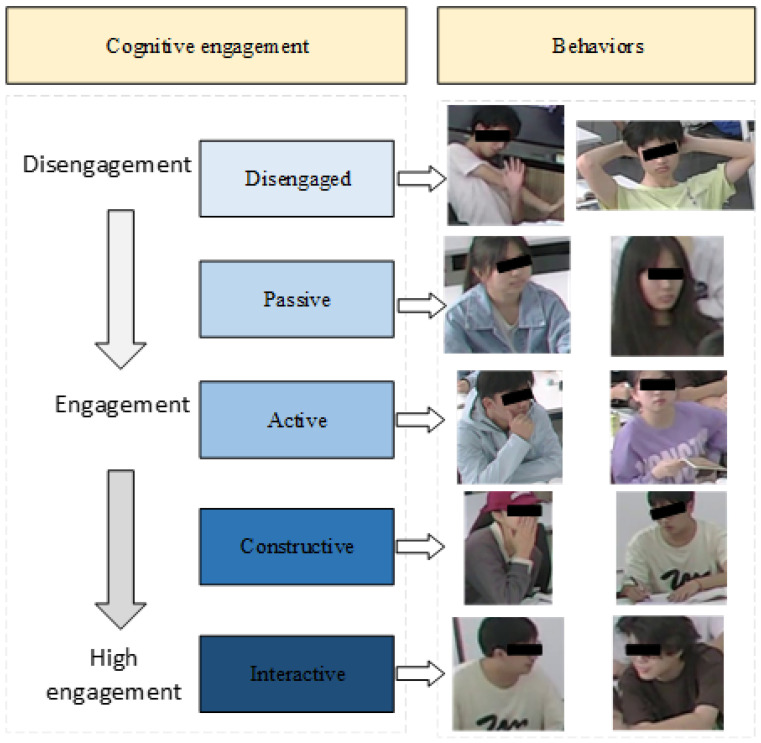
The ICAPD framework for labeling. It includes five classes of cognitive engagement and their corresponding behaviors.

As shown in Figure 5, the generated images were annotated using the LabelImg data annotation tool. Moreover, based on the ICAPD framework [9], the locations and behaviors of the individual students were precisely marked with bounding boxes (seen in Figure 6).

We further defined different degrees of cognitive engagement in university classrooms, referring to the work of [9]. The defined degrees are presented in Table 2. Each student’s location is delineated by the smallest possible rectangular bounding box, thereby ensuring that the enclosing boundary contains a minimal amount of the surrounding background. The annotation details include the folder name, filename, path, source, size, and multiple objects. These annotated files were saved with an XML extension. The SCE dataset contains very few instances, with annotations in each image being densely packed. This characteristic poses a considerable challenge in accurately capturing the nuances of student behavior for automatic detection.

After labeling according to the ICAPD framework, the SCE dataset generated in this experiment contains 5665 images with a total of 154,776 objects. The dataset’s structure is configured in accordance with the COCO dataset schema. Before data augmentation, 3880, 5072, 12,011, 41,922, and 91,891 samples were in the disengaged, constructive, active, passive, and interactive categories, respectively. The composition of the training and testing samples is shown below (see Table 3).

### 4.2. Experimental Setting

This study’s experimental environment consisted of training the models on an NVIDIA GeForce RTX 3090 (NVIDIA, Santa Clara, CA, USA) and leveraging the GPU drivers compatible with Ubuntu 22.04. And the utilized environment comprised Python 3.11.7 and torch 2.1.2 with cuda 12.1 support.

All experiments within this section were configured to undergo a training regimen comprising 300 epochs. The training was stopped early if the average accuracy did not improve significantly after 50 epochs. The YOLOv8n model, a component of the YOLOv8 series, was used to train with a batch size of 128, which was constrained by the GPU’s memory capacity. The learning rate utilized during the model’s training phase was set to 0.01, with an SGD momentum of 0.937 and an optimizer weight decay of 0.0005. All other training parameters were maintained at their default values as specified by the YOLOv8n network architecture.

To comprehensively assess the proposed model’s performance, we utilized precision (P), recall (R), F1 score (F1), mean average precision when IoU is 0.5 (mAP50), and mean average precision when IoU is 0.5 to 0.95 (mAP50-95) to measure the model’s accuracy and evaluate the object detection results on the SCE dataset.

### 4.3. Training Procedures

**Figure 7:**
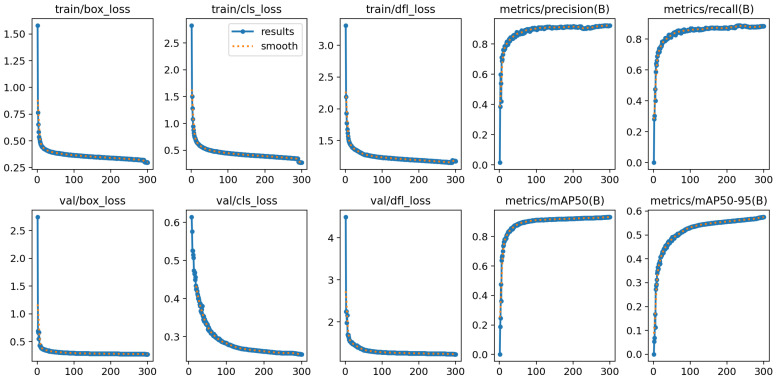
Performance for the OE-YOLOv8n model in the training procedure on the SCE dataset.

As shown in Figure 7, the initial three columns depict the training’s time progression along the X-axis and the corresponding loss values along the Y-axis. The overall loss values continue to decrease as training progresses and eventually stabilizes. The experimental results show that the OE-YOLOv8n model demonstrates robust fitting performance, high stability, and a high level of accuracy. The last two columns represent the P, R, mAP50, and mAP50-95 curves, and the X-axis represents the training time. It can be observed that the curves’ values gradually approach 1, indicating the effectiveness of the OE-YOLOv8n model. In all, when the epoch is 300, the overall training effect of our method is ideal. Therefore, the weight file of the acquired detection model is saved, and the test set is utilized for the assessment of the model’s efficacy.

### 4.4. Experimental Comparison

#### 4.4.1. Comparison with Baseline

**Figure 8:**
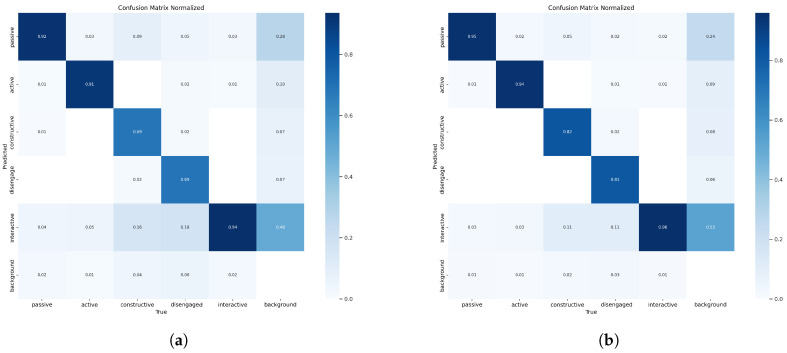
Confusion matrices of the YOLOv8n and OE-YOLOv8n methods on the SCE dataset. The rows correspond to the true labels, the columns correspond to the predicted categories, and the diagonal entries correspond to the accuracy of correct predictions. (**a**) YOLOv8n; (**b**) OE-YOLOv8n.

Figure 8 displays the confusion matrix for the OE-YOLOv8n model and depicts its prediction accuracy for the five distinct categories of student cognitive engagement within the SCE dataset. Additionally, it illustrates the relationship between the predictions. The matrix clearly demonstrates that our method attains high accuracy for each category.

The efficacy of the proposed SCE detection method was substantiated through a comparative analysis involving five distinct methods. These baseline approaches include Faster R-CNN and SSD; YOLO families YOLOv5n and YOLOv8n; and a variant of YOLOv8n, simAM-YOLOv8n. These experiments were conducted on the test dataset we established. The corresponding experimental results are displayed in Table 4.

It can be seen that the P, R, F1, mAP50, and mAP50-90 of OE-YOLOv8n are higher than those of other algorithms. The performance of the OE-YOLOv8n model surpasses that of the two-stage detection model (i.e., Faster R-CNN), indicating that the deep cognitive engagement features extracted by OE-YOLOv8n are more effective. Compared to the single-stage model (i.e., SSD), the proposed OE-YOLOv8n model still demonstrates comparability in F1 score, mAP, etc. The comparison within the YOLO family reveals that the architecture of YOLOv8n is superior, leading to better results using the proposed improvement strategies after YOLOv8n. The experimental results with different improvement strategies suggest that our enhancement (i.e., data-enhanced and improved-loss) is more suitable for cognitive engagement detection tasks. After data augmentation and IMPDIoU loss improvement, the OE-YOLOv8n method achieved significant performance enhancement in the SCE dataset.

#### 4.4.2. Ablation Experiments

In order to further understand the significance of the two improved modules, the OE-YOLOv8n model was run on the SCE dataset by removing the Mosaic and Cutout data augmentation and the IMPDIoU loss function. Then, the evaluation values were set on the SCE dataset. The results of the ablation experiments are summarized in Table 5.

YOLOv8n denotes the original model, and L-YOLOv8n denotes the YOLOv8n model with an IMPDIoU loss function. The last row refers to the improved network in this paper. The last five columns show the model’s P, R, F1, mAP50, and mAP50-95 values without one or two of these modules.

From the table, it is discernible that the P, R, F1, mAP50, and mAP50-95 values experience a precipitous decrease when any one or two of the modules are omitted. This attests to our conjecture that a more balanced distribution of cognitive engagement samples and a more precise estimation of IoU values are indispensable for the accurate assessment of cognitive engagement levels. Due to the proposed IMPDIoU loss improvement strategy employed in L-YOLOv8n, the L-YOLOv8n method exhibits higher mAP values at different IoU thresholds. OE-YOLOv8n outperforms YOLOv8n the most in terms of the P value (improved by 6.4%), indicating that the proposed OE-YOLOv8n model can maintain high accuracy in recognizing students and their cognitive engagement. Furthermore, the OE-YOLOv8n method exhibits higher F1 values than the L-YOLOv8n method. This indicates that our proposed data augmentation strategy is effective, enabling more accurate computation of high-order cognitive engagement (i.e., interactive) with few samples.

#### 4.4.3. Validity of the Method

**Figure 9:**
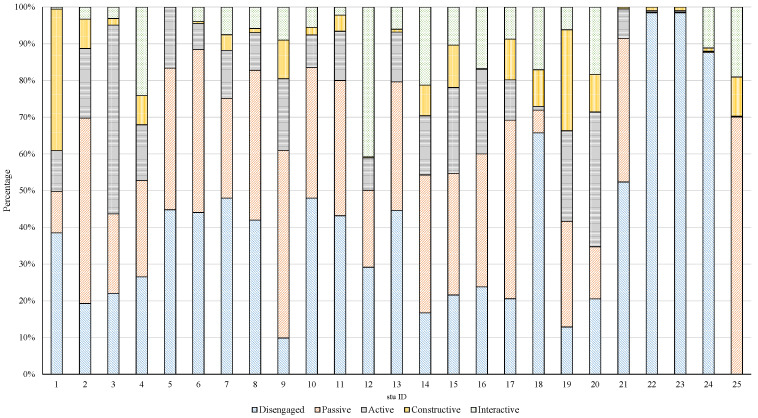
The results of detecting students’ cognitive engagement in a class. The X-axis represents the sequence of student IDs and the Y-axis represents the proportion of each state in the video.

To validate the effectiveness of the algorithm in detecting cognitive engagement, experiments were conducted using class videos. The OE-YOLOv8n model in track mode was employed to detect cognitive engagement in video data captured every 3 s/frame. Each student was assigned a fixed ID, and the occurrences of disengaged, passive, active, constructive, and interactive states for each student in the video were recorded. The data for each state category were normalized using min–max scaling. The results are shown in Figure 9. It was observed that (1) the cognitive engagement states detected by the OE-YOLOv8n model were not consistently associated with specific individuals. This indicates that the cognitive engagement states exhibited by students are not always fixed. (2) There were no identical cognitive engagement patterns across the entire video, emphasizing individual differences. Therefore, the model does not focus on detecting individuals but rather on capturing various cognitive engagement features.

## 5. Conclusions

This investigation introduces OE-YOLOv8n as a solution to the challenges encountered in the detection of cognitive engagement within real-world classroom environments. On the one hand, the YOLOv8n model and the improved IMPDIoU loss function are employed to address the occlusion issue. The IMPDIoU directly minimizes the distance between the top-left and bottom-right points of the predicted bounding box and the ground truth bounding box. Additionally, a scale factor is integrated to regulate the scale of auxiliary bounding boxes. On the other hand, the Mosaic method and Cutout method are combined to augment the SCE dataset. As a result, even the difficult-to-recognize and infrequently occurring categories in real classrooms can receive significant attention from the improved model. The experimental results indicate that OE-YOLOv8n yields a substantial enhancement in detection efficacy. Furthermore, ablation experiments were conducted to corroborate the efficacy of the various modules. In the subsequent stages, we intend to integrate additional engagement cues, such as heart rate and sweat sensors, to further enrich our investigation.

## Figures and Tables

**Table 1:** The performance of OE-YOLOv8n with different ratios of IMPDIoU loss against the standard MPDIoU loss. The bold represents the best result.

Ratio	P	R	F1	mAP50	mAP50-95
MPDIoU	0.901	0.866	0.883	0.917	0.559
IMPDIoU (ration = 0.6)	0.913	0.879	0.896	0.924	0.565
IMPDIoU (ration = 0.8)	0.910	0.887	0.898	0.928	0.567
IMPDIoU (ration = 1.0)	**0.921**	0.885	0.903	0.932	0.575
IMPDIoU (ration = 1.2)	0.920	0.885	0.902	0.934	**0.576**
**IMPDIoU (ration = 1.4)**	0.918	**0.891**	**0.904**	**0.935**	0.573

**Table 2:** Degrees of cognitive engagement of the students in a classroom.

Classes	Behaviors	Example Behaviors
Disengaged	Behavior unrelated to learning	Yawning, drinking water, lying on the desk, sleeping, looking out the window, playing on a phone/computer
Passive	Sitting silently	Seated with a static posture (no movement of the head, hands, or body)
Active	Thinking and operating learning materials	Pointing to materials, underlining sentences, taking out tools, scratching the head, hands on the face or head
Constructive	Generating and expressing new ideas	Taking notes, raising hands, drawing on paper
Interactive	Dialogue with teachers or students	Standing up to talk to teachers, turning the body and talking to peers, applauding mates, clapping hands, patting others

**Table 3:** The scale of the SCE dataset.

	Training	Testing	Total
Passive	33,613	8309	41,922
Active	9481	2530	12,011
Constructive	4071	1001	5072
Disengaged	3107	773	3880
Interactive	73,516	18,375	91,891
total	123,788	30,988	154,776

**Table 4:** Comparison of methods and our OE-YOLOv8n results on SCE dataset. The bold represents the best result.

Method	P	R	F1	mAP50	mAP50-95
Faster R-CNN	0.815	0.637	0.715	0.820	0.504
SSD	0.831	0.573	0.678	0.833	0.479
YOLOv5n	0.849	0.816	0.832	0.852	0.508
YOLOv8n	0.853	0.823	0.838	0.866	0.549
simAM-YOLOv8n	0.861	0.832	0.846	0.875	0.548
**OE-YOLOv8n**	**0.918**	**0.891**	**0.904**	**0.935**	**0.573**

**Table 5:** Ablation experiments on SCE dataset. The bold represents the best result.

Method	P	R	F1	mAP50	mAP50-95
YOLOv8n	0.853	0.823	0.838	0.866	0.549
L-YOLOv8n	0.870	0.841	0.855	0.880	0.520
**OE-YOLOv8n**	**0.918**	**0.891**	**0.904**	**0.935**	**0.573**

## Data Availability

Data are unavailable due to privacy or ethical restrictions.

## Abbreviations

The following abbreviations are used in this manuscript:

OE-YOLOv8n	Object-Enhanced–You Only Look Once version 8 nano.
IMPDIoU	Inner Minimum Point Distance Intersection over Union.
SCE	Students’ Cognitive Engagement.
ICAPD	interactive, constructive, active, passive, and disengaged.
BCE	Binary Cross-Entropy.
DFL-CIoU	Distribution Focal Loss and Complete Intersection over Union.
